# Endo/Lysosomal-Escapable Lipid Nanoparticle Platforms for Enhancing mRNA Delivery in Cancer Therapy

**DOI:** 10.3390/pharmaceutics17070803

**Published:** 2025-06-20

**Authors:** Jiapeng Wang, Renjie Chen, Yongyi Xie, Xuanting Qin, You Zhou, Chuanshan Xu

**Affiliations:** 1 Department of Medical Imageology, The Second Clinical College of Guangzhou Medical University, Guangzhou 511436, China; 2022113088@stu.gzhmu.edu.cn; 2 State Key Laboratory of Respiratory Disease, School of Pharmaceutical Sciences, Guangzhou Medical University, Guangzhou 511436, China; 2022123041@stu.gzhmu.edu.cn (R.C.); 2022210636@stu.gzhmu.edu.cn (Y.X.); 2023123017@stu.gzhmu.edu.cn (X.Q.)

**Keywords:** lipid nanoparticle, endo/lysosomal escape, mRNA delivery, gene therapy

## Abstract

mRNA-based drug development is revolutionizing tumor therapies by enabling precise cancer immunotherapy, tumor suppressor gene restoration, and genome editing. However, the success of mRNA therapies hinges on efficient delivery systems that can protect mRNA from degradation and facilitate its release into the cytoplasm for translation. Despite the emergence of lipid nanoparticles (LNPs) as a clinically advanced platform for mRNA delivery, the efficiency of endo/lysosomal escape still represents a substantial bottleneck. Here, we summarize the intracellular fate of mRNA-loaded LNPs, focusing on their internalization pathways and processing within the endo-lysosomal system. We also discuss the impact of endo-lysosomal processes on mRNA delivery and explore potential strategies to improve mRNA escape from endo-lysosomal compartments. This review focuses on molecular engineering strategies to enhance LNP-mediated endo/lysosomal escape by optimizing lipid composition, including ionizable lipids, helper lipids, cholesterol, and PEGylated lipids. Additionally, ancillary enhancement strategies such as surface coating and shape management are discussed. By comprehensively integrating mechanistic insights into the journey of LNPs within the endo-lysosome system and recent advances in lipid chemistry, this review offers valuable inspiration for advancing mRNA-based cancer therapies by enabling robust protein expression.

## 1. Introduction

Cancer is a leading cause of death affecting millions of people worldwide. Conventional therapies including surgery, radiotherapy, and chemotherapy often cause severe side effects due to their low specificity. Gene therapy is a transformative approach in modern medicine that leverages genetic modification to treat diseases by introducing, altering, or replacing genetic material in a patient’s cells [1]. Distinguished from conventional therapies, gene therapy offers durable or even curative outcomes by directly targeting the molecular underpinnings of pathology through editing, supplementing, or silencing disease-causing genes. This strategy holds immense potential in addressing a wide range of conditions, particularly those with a genetic basis, including previously intractable cancers [2]. A key advantage of gene therapy lies in the versatility of nucleic acid drug formats, which include plasmid DNA, viral vectors, small interfering RNA, and mRNA or ribonucleoprotein (RNP) complexes. Among these modalities, mRNA-based therapies have demonstrated significant potential in both therapeutic and preventive applications, as they provide a platform to directly modulate cellular processes by delivering genetic instructions for transiently producing proteins, which avoids genomic integration [3].

The success of mRNA-LNP vaccines against COVID-19 underscored their rapid manufacturability, scalability, and efficacy in eliciting robust immune responses [4]. Beyond infectious disease vaccines, mRNA technologies are being explored for diverse applications, including vaccines against HIV, tuberculosis, and malaria, as well as protein replacement in monogenic disorders like methylmalonic acidemia and cystic fibrosis [5]. mRNA-encoded gene-editing tools such as CRISPR-Cas9 are being used for precise genomic modifications, with applications in treating amyloidosis and lowering LDL cholesterol through PCSK9 editing [6,7].

Recent efforts have focused on harnessing mRNA for cancer therapy. For instance, mRNA vaccines encoding tumor antigens or neoantigens can elicit potent immune responses against cancer cells [8]. Clinical trials are currently evaluating their efficacy and safety in treating cancers such as melanoma, colorectal cancer, and pancreatic cancer [9]. Cytokines such as IL-2 and IL-12 play crucial roles in activating T cells and natural killer (NK) cells [10,11]. In contrast, IFNα directly inhibits tumor cell proliferation and enhances antigen presentation, collectively contributing to a more effective antitumor response [12]. mRNA encoding these cytokines has also been developed to elicit antitumor immunity by enabling cytokine expression. Other strategies involve delivering mRNA-encoding tumor suppressors like TP53 and PTEN to restore their tumor-suppressive functions and sensitize cancer cells to conventional therapies [13,14]. Moreover, mRNA-based genome-editing tools, such as CRISPR-Cas9, offer the potential for precise genetic modifications within cancer cells, disrupting oncogenic pathways and enhancing therapeutic outcomes [15]. Furthermore, mRNA-encoding chimeric antigen receptors (CARs) or T cell receptors (TCRs) enable the engineering of T cells for targeted cancer cell elimination [16]. Collectively, these diverse applications highlight mRNA’s versatility in addressing key challenges in oncology [17].

Although mRNA-based therapy shows great promise, mRNA stability and translation efficiency remain significant challenges. To enhance the stability and expression efficiency of mRNA while reducing its immunogenicity, several key strategies have been developed [18]. Chemical modifications include the incorporation of modified nucleosides such as pseudo-uridine, N6-methyladenosine, 5-methylcytidine, and 5-methyluridine, which can mimic natural RNA modifications to evade immune detection and improve stability and translation efficiency. Optimizing the 5′ cap structure with modified cap analogs and adjusting the poly(A) tail length and composition further enhances mRNA stability and protein expression [19]. Sequence optimization through enriching the GC content, optimizing codon usage to match the host cell’s preferences, and modifying the 5′ and 3′ untranslated regions (UTRs) to incorporate regulatory elements can also enhance mRNA stability and translation [20].

Beyond chemical modifications, advanced mRNA formats such as trans-amplifying mRNA (taRNA) and circular mRNA (circRNA) offer innovative solutions. Self-amplifying mRNA contains viral replicase genes, enabling self-amplification, leading to prolonged protein expression [21]. Trans-amplifying mRNA (taRNA) separates the replicase and the protein-of-interest into two shorter RNAs, enhancing safety [22]. Circular mRNA (circRNA) forms a closed loop, conferring high resistance to exonucleases, superior stability, and sustained protein expression [23]. These strategies collectively enhance mRNA’s therapeutic potential by improving stability, reducing immunogenicity, and optimizing expression efficiency.

**Figure 1:**
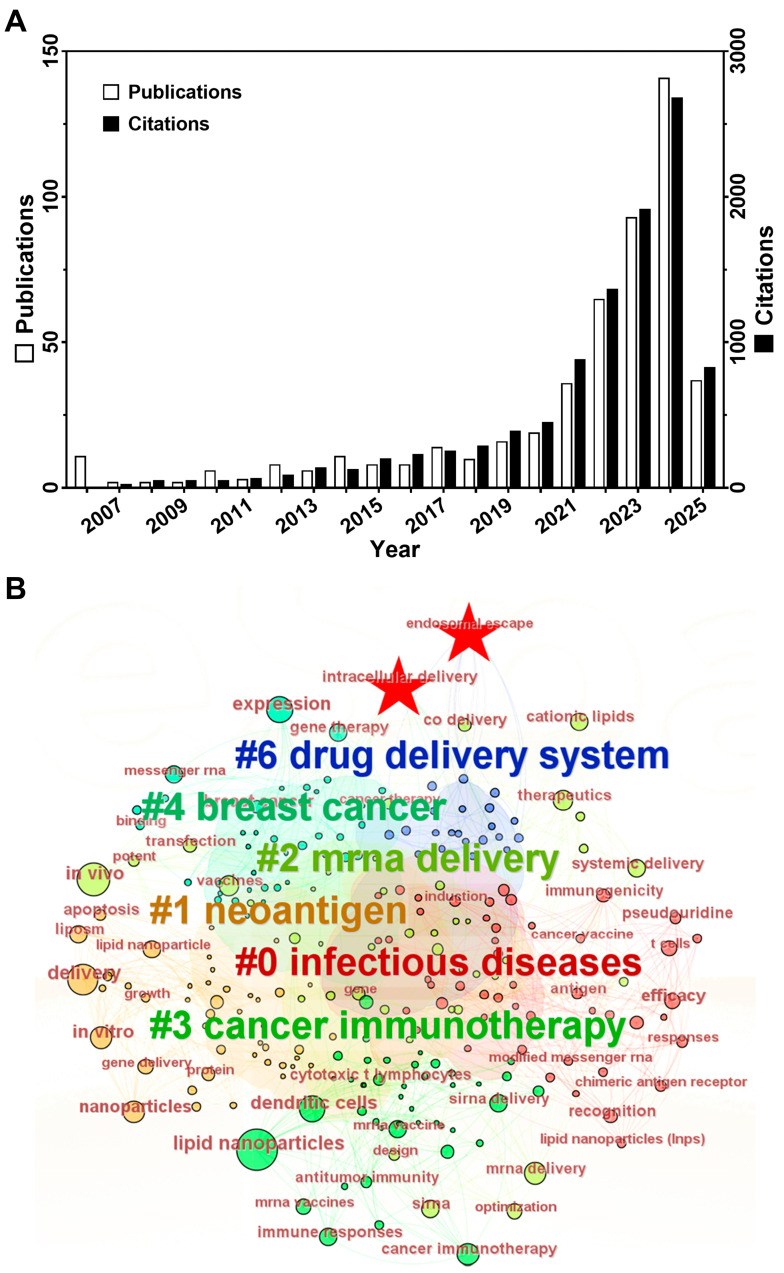
The basic bibliometric analysis of LNPs in mRNA delivery for cancer therapy. (**A**) The annual distributions of publications and their citations. Data were collected using the search query (TS = (Cancer OR Tumor OR Carcinoma) AND AB = (“mRNA” OR “Circular RNA”) AND AB = (Deliver*) NOT AB = (siRNA) AND TS = (“lipid nanoparticle*” OR “LNP” OR “liposome*”)) in Web of Science core collection from 2006 to 2025, and the histogram was created by using GraphPad Prism 9. (**B**) The main topics in this field analyzed by CiteSpace Version 6.3.R1. All keywords were extracted from the abstract of hit publications identified in A. Each circle represents a keyword, and its color matches the color of the cluster it belongs to (enlarged words are guided by #). Specifically, the two keywords related to endosomal/lysosomal escape, ‘intracellular delivery’ and ‘endosomal escape’, are highlighted with stars.

Despite improvements from chemical modifications, mRNA remains vulnerable to extracellular ribonucleases and rapid systemic clearance. Delivery carriers address these limitations by encapsulating mRNA to protect it from nucleases and enabling precise tissue/cell targeting through surface modifications like antibodies and ligands. This minimizes off-target effects while ensuring that therapeutic payloads reach specific sites [24]. Viral vectors, such as adenovirus and adeno-associated virus, offer high transfection efficiency and long-term expression, making them suitable for hereditary disease gene therapy or CAR-T cell engineering. However, pre-existing immunity, insertional mutagenesis risks, and genomic integration safety concerns limit their widespread applications, especially for repeated dosing [25]. In contrast, non-viral systems like lipid nanoparticles (LNPs) provide distinct advantages. They consist of ionizable lipids, cholesterol, helper lipids, and PEGylated lipids, which together enhance mRNA stability and reduce toxicity. The design flexibility allows engineering for specific tissue/cell targeting, making them highly versatile [26]. Additionally, as the precursor to LNP technology, liposomes share nearly identical material compositions and similar physical properties with LNPs. In numerous studies, a clear distinction between liposomes and LNPs is often not strictly made. Therefore, we have elected to classify liposomes under the broader category of LNPs in this review. Owing to the rapid production capabilities, adaptability, and improved safety profile, LNPs have become the most widely used tool in mRNA delivery [27]. Figure 1A illustrates the development and research trends in LNP-based mRNA delivery over the past two decades. A Web of Science core collection from 2006 to 2025 using the query: TS = (Cancer OR Tumor OR Carcinoma) AND AB = (“mRNA” OR “Circular RNA”) AND AB = (Deliver*) NOT AB = (siRNA) AND TS = (“lipid nanoparticle*” OR “LNP” OR “liposome*”) yielded 498 research articles with 10133 cumulative citations. The steady increase in publications and citations reflects growing momentum and development in this area.

LNPs possess unique properties that can be tailored for tissue-specific delivery by adjusting the lipid components ratio, enhancing therapeutic efficacy while reducing off-target effects. Recent advances include novel ionizable lipids and targeting ligands to improve specificity [28]. For instance, selective organ targeting (SORT) molecules enable tissue-specific mRNA delivery to the liver, spleen, and lungs, respectively. Biodegradable lipids (e.g., SS-OP) address LNP hepatotoxicity by enhancing metabolic clearance and reducing long-term toxicity [29]. Microfluidic techniques further optimize LNP synthesis, improving batch consistency and encapsulation efficiency [30]. Beyond vaccines, LNPs enable groundbreaking applications, like in vivo CRISPR-Cas9 gene editing, mRNA-based protein replacement therapy, and hybrid systems co-delivering nucleic acids with small molecules for combination therapies [31]. Owing to their adaptable, modular design for diverse therapeutics, LNPs represent the gold standard in gene delivery.

The efficient release of mRNA from endosomes into the cytosol is essential for functional expression and subsequent therapeutic efficacy. However, achieving effective endosomal and lysosomal escape remains a formidable challenge for LNP-mediated mRNA delivery [32]. After cell uptake, mRNA is trafficked to endosomes and lysosomes, where it risks extrusion via endocytic recycling or degradation by the acidic environment and lysosomal enzymes [33]. Despite significant advancements, the limited endosomal escape efficiency of nucleic acid (~1–2%) persists as a critical bottleneck, as demonstrated by quantitative fluorescence imaging and electron microscopy [34]. This inefficiency stems from the endosomal system’s complex, dynamic maturation stages and degradation pathways. Optimizing this process is thus vital for enhancing mRNA therapies. Bibliometric analysis of publications regarding LNPs for mRNA delivery in cancer using CiteSpace (version 6.3.R1) reveals insufficient focus on endosomal/lysosomal escape over the past decade. While keywords like “intracellular delivery” and “endosomal escape” appear (Figure 1B), their prominence remains low relative to this mechanism’s importance.

While several reviews have discussed the development of mRNA and endo/lysosomal escape-enabled delivery vehicles in cancer, there remains a notable gap in the literature with no comprehensive exploration dedicated to this specific domain, despite the pivotal role of LNPs in mRNA delivery [35,36]. In this review, we aim to bridge this gap by summarizing endo/lysosomal-escapable lipid nanoparticle platforms for mRNA delivery and showcasing how LNPs facilitate the endo/lysosomal escape of mRNA. We place particular emphasis on the intrinsic properties of LNPs that govern this escape capability. Our discussion is poised to provide insights that will inform the further development of LNPs with superior endo/lysosomal escape properties for cancer therapy.

## 2. The Fate of mRNA-Loaded LNP

### 2.1. Pathway of mRNA-Loaded LNP Internalization

The process by which LNPs are taken up by cells profoundly influences the subsequent escape of mRNA. The intracellular journey of mRNA-loaded LNPs begins with cellular internalization via endocytosis, governed by diverse pathways. These pathways, including phagocytosis, macropinocytosis, clathrin-mediated endocytosis (CME), and caveolae-mediated endocytosis (CVME), not only determine the initial uptake efficiency but also profoundly influence subsequent trafficking dynamics and opportunities for endosomal escape [37]. As thoroughly reviewed by Manzanares, D., and Ceña, V. carrier properties, including size, charge, shape, and rigidity, are the main influences on their endocytic pathways. Smaller carriers (below 200 nm) are predominantly internalized via CME or CVME, while larger carriers (above 200 nm) are more likely to be internalized by macropinocytosis or phagocytosis. Cationic carriers tend to enter cells through CME due to interactions with negatively charged cell membranes, whereas anionic carriers are more often associated with CVME. Additionally, spherical and rigid carriers are often internalized by CME, while elongated or flexible carriers are internalized via macropinocytosis [38]. Understanding these entry mechanisms explains why mRNA-loaded LNPs predominantly accumulate in endo-lysosomal compartments and how their escape kinetics are constrained by the vesicular transport machinery.

Phagocytosis is a process primarily utilized by macrophages, neutrophils, and dendritic cells to engulf large particles, including bacteria, apoptotic cells, and nanoparticles [39]. This process is characterized by the ligand-receptor binding followed by zipper-like engulfment into a phagosome. Robust actin polymerization drives membrane encapsulation, forming large, stable vesicles [40]. This highly efficient process is regulated by pathways like the CD47-SIRPαaxis [41]. While traditionally associated with large particles, relatively recent evidence confirms that phagocytosis internalizes smaller NPs, challenging the notion that only particles larger than 500 nm can be phagocytosed [42,43].

Macropinocytosis, in contrast, is a process that mediates the bulk uptake of extracellular fluid and particles via large, fluid-filled macropinosomes [44]. Triggered by growth factors or oncogenic signaling, it involves membrane ruffling without requiring specific particle-receptor binding. Macropinosomes are heterogeneous in size and can fuse with early endosomes, eventually maturing into late endosomes and lysosomes. Though sharing actin dynamics with phagocytosis, macropinosomes are distinct by their fluid-phase uptake mechanism [45].

CME is responsible for the selective uptake of smaller cargo, typically less than 100 nm in diameter [46]. CME involves clathrin-coated pits (CCPs) formation, invagination, and dynamin-mediated scission to form clathrin-coated vesicles (CCVs). CCVs rapidly fuse with early endosomes. Efficiency depends on receptor density, ligand affinity, and the presence of specific signaling pathways. While well-characterized, precise mechanisms of clathrin coat assembly/disassembly and actin’s role remain incompletely understood [47]. Despite its specificity for small cargo, CME can also accommodate larger particles, such as certain viruses and engineered NPs, through mechanisms that involve receptor clustering and the enlargement of CCPs [48,49].

CVME internalizes cargo via small, cholesterol- and caveolin proteins-rich plasma membrane invaginations, abundant in endothelial cells, adipocytes, and smooth muscle cells. CVME is thought to play a role in the uptake of certain lipids and small molecules, although its contribution to NPs uptake remains a subject of debate. Unlike phagocytosis and macropinocytosis, it requires minimal actin polymerization or signaling [50]. In particular, the caveosome can traffic cargo to the Golgi and/or endoplasmic reticulum, thus bypassing subsequent endosome and lysosomal processes [51].

Generally speaking, LNPs’ internalization depends on their composition and the cell type involved. Dobrowolski, C. and his colleagues revealed significant cell subtype heterogeneity in LNP uptake efficiency, driven by transcriptional differences [52]. CME is favored due to LNP–receptor interactions, while CVME can associate with lipid rafts. Digiacomo, L., et al. showed that PEGylated LNPs possessed a stronger preference for CME and CVME pathways, whereas plain LNPs favor macropinocytosis [53]. In another study, Lili, C. et al. proved that the expression of the standard LNPs-loaded mRNA could be inhibited by 40% through CME inhibitor [54]. The study also demonstrated that some receptors located in caveolae, such as insulin receptors and epidermal growth factor receptors, could mediate CVME [55]. In Cav1^−/−^ mice, the delivery of LNP to Kupffer cells, the liver-resident macrophages, is dramatically reduced by up to 93% [56]. In some cases, LNPs could also enter cells via macropinocytosis. Sahay, G. and his colleagues found that cationic LNPs are primarily internalized via Cdc42-dependent macropinocytosis, a process regulated by proton pumps (VoATPase), mTOR, and cathepsins. This conclusion was validated by inhibitor and knockdown studies [57]. Consistently, a study revealed that cholesterol depletion but not inhibitors of CME or CVME could significantly reduce the rapid uptake of lipid nanocapsules (LNCs), indicating a vital role of macropinocytosis in its internalization [58].

In summary, phagocytosis, macropinocytosis, CME, and CVME exhibit distinct mechanisms guiding LNPs into the endosome, where the fate of mRNA will be determined to undergo functional cytoplasmic release, the process of endocytic cycling, or enzymatic degradation. Understanding the subtle differences among these pathways is important for optimizing LNP design and delivery efficacy. Future research elucidating precise nanomaterial uptake mechanisms will help clarify the metabolic routes and fate of internalized LNPs.

### 2.2. Processing of mRNA-Loaded LNP in Endo-Lysosome System

As the ensuing process of cellular internalization, mRNA-loaded LNPs are usually transmitted into the endosomal–lysosomal transport system, which is a highly orchestrated network of dynamic, interconnected vesicular structures that maintain cellular homeostasis by regulating the degradation and recycling of internalized cargo [59]. This process comprises distinct compartments, including early endosomes, recycling endosomes, late endosomes, and lysosomes. Coordinated changes in membrane composition, protein sorting, and progressive acidification dictate the ultimate fate of internalized cargo [60].

**Figure 2:**
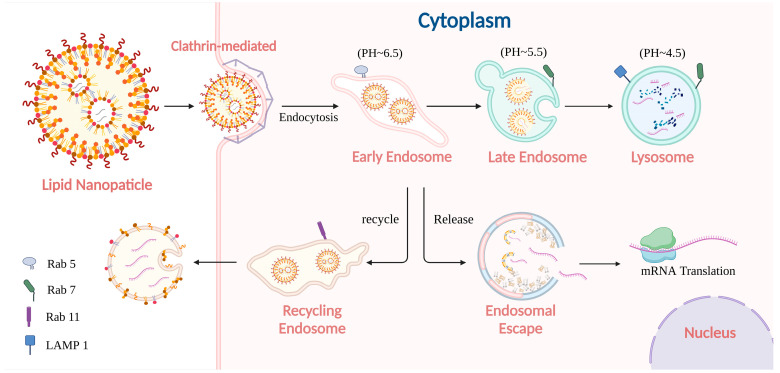
A typical intracellular journey of LNP-encapsulated mRNA in application. The mRNA-loaded LNP is introduced into early endosomes after being internalized through CME, from which some are recycled back to the extracellular space. A portion of the mRNA successfully escapes to the cytoplasm in the same period, achieving its expected translation. Meanwhile, another fraction of the LNPs progresses through maturation into late endosomes and eventually into lysosomes, where they are subjected to degradation.

As depicted in Figure 2, presuming that the internalization of LNPs was initiated through CME, clathrin-coated pits on the plasma membrane bud off to form vesicles. These vesicles fuse with early endosomes, characterized by a slightly acidic pH (around 6.5) and the presence of Rab5, a small GTPase regulating early endosomal functions [61]. Early endosomes serve as a primary sorting hub where receptor–ligand dissociation occurs. Receptors typically recycle back to the plasma membrane, while dissociated ligands and other cargo are sorted for transport toward late endosomes or lysosomes for degradation [62,63]. This sorting process is governed by Rab GTPases and the endosomal sorting complexes required for transport (ESCRT) machinery [64].

As the endosome matures, key transformations occur. One of the most notable changes is the intraluminal vesicle via inward budding, facilitated by the ESCRT machinery. This sorts ubiquitinated proteins into vesicles, forming multivesicular bodies (MVBs) crucial for the degradation and signaling regulation [65]. Another aspect is the progressive acidification of the lumen from early endosomes (pH ~6.5) to late endosomes (pH ~5.5), and finally to lysosomes (pH ~4.5) [66]. This acidification is primarily driven by the vacuolar ATPase proton pump that actively transports protons into the endosomal lumen [67]. The acidic environment is indispensable for the function of hydrolases and other enzymes involved in the degradation. To counterbalance the positive charge, chloride channels passively transport chloride ions into the lumen to counterbalance proton influx, maintaining the electrochemical gradient [68].

Early endosomes relocate from the cell periphery toward the microtubule organizing center as they mature into late endosomes. Dynein motors mediate the movement along microtubules, positioning endosomes near lysosomes to enhance degradation efficiency [69]. A hallmark of endosome maturation is the switch in Rab proteins. Rab5, associated with early endosomes, is replaced by Rab7, which is characteristic of late endosomes [70]. This conversion drives endosomal formation, regulating fusion with lysosomes and altering lipid composition and membrane curvature [71].

The fate of cargo within the endosomal–lysosomal system is determined by two opposing sorting systems: the ESCRT machinery and the retromer complex [72]. The ESCRT machinery sorts ubiquitinated proteins into intraluminal vesicles for lysosomal degradation. It comprises four distinct complexes: ESCRT-0 recognizes and clusters ubiquitinated proteins on the endosomal membrane, while ESCRT-I and ESCRT-II further concentrate and sort them. ESCRT-III drives membrane invagination and scission while deubiquitinating cargo [73]. Conversely, the retromer complex retrieves specific cargo from early endosomes to the trans-Golgi network or plasma membrane, preventing degradation. Its cargo-selective complex, composed of Vps35, Vps29, and Vps26, recruited by sorting nexins (SNXs), packages recyclable cargo into tubulo-vesicular carriers. It was summarized that cargo destined for lysosomal degradation is sorted into intraluminal vesicles (ILVs) within late endosomes, while recyclable cargo is packaged into tubulo-vesicular transport carriers returning to the plasma membrane or the secretory pathway. The sorting pathways determine whether cargo undergoes recycling or degradation [74].

### 2.3. The Impact of Endo-Lysosome Process on mRNA Delivery

Once endocytosed, LNP-encapsulated mRNA must achieve endosomal escape to reach the cytoplasm for translation; therefore, endocytic recycling and enzymatic degradation undoubtedly compromise its biological function. Endocytic recycling typically occurs during the endosomal phase, where the endosomal membrane redistributes nucleic acid cargo via interactions with LNPs. Marco Maugeri et al. reported that the endocytosed LNPs localize to endosomal-derived extracellular vesicles, whose membranes exhibit significantly altered composition compared to the original LNPs, indicating intense membrane component exchange during endosomal transit [75]. Studies modeling early and late endosomal membranes revealed that LNP–endosomal membrane interactions are strongly pH-dependent, with maximal interactions occurring at acidic pH (≤6.5). Under these conditions, ionizable lipids in LNPs become protonated, which allows them to interact with anionic lipids present in the model endosomal membrane, facilitating lipid insertion into the endosome and the escape of the nucleic acids. mRNA-loaded LNPs show stronger interactions than Poly(A)-loaded LNPs due to differences in charge density and complex stability, while the endosomal maturation stage has minimal impact on the interaction, confirming pH as the key determinant in LNP-endosomal membrane interactions [76]. The sorting of internalized LNPs or the mRNA components into recycling endosomes leads to their cellular efflux. Research on siRNA delivery by LNPs indicates that approximately 70% of internalized siRNA undergoes exocytosis via late endosome-derived tubulo-vesicular carriers. This loss is regulated by Niemann Pick type C1 (NPC1), a recycling pathway protein, while NPC1knockout enhances LNP retention in late endosomes/lysosomes and significantly increases gene silencing efficiency [75]. Similarly, NPC1-deficiency enhances mRNA transfection efficiency, confirming that reduced endosomal recycling improves intracellular availability and mRNA delivery [77].

Except for those re-exocytosed, the remaining mRNAs undergo subsequent intracellular processing. A portion successfully escapes from the endosomes/lysosomes into the cytoplasm and exerts biological functions. Single-molecule fluorescence in situ hybridization and super-resolution microscopy visualize mRNA escape from the endosome at the nanoscale, revealing that Rab11-positive recycling endosomes are key escape sites. This escape process is correlated with endosomal acidification because proper acidification is significant to mRNA release from LNPs. Additionally, endosome size impacts escape efficiency, as smaller endosomes (marked by EEA1 and APPL1) are more conducive to mRNA escape compared to the larger, arrested endosomes. Notably, mRNA diffusion from recycling endosome tips or along the tubular structures has been directly observed [78]. Escape before lysosomal maturation is thus essential yet remains a major delivery barrier. Conventional LNPs deliver only 1–4% of mRNA to the cytoplasm, as most cargo is trapped and subsequently degraded in lysosomes. This low endosomal escape rate severely limits therapeutic applications [79]. Notably, newer ionizable lipids achieve nearly 15% endosomal release efficiency, surpassing the 2–5% rate of early-generation MC3 siRNA-LNPs [80].

Finally, the remaining RNA in late endosomes is sorted into ILVs and then fuses with lysosomes to form endo-lysosomes. The lysosomes, which are recognized by LAMP1, have an acidic environment (pH around 4.5–5.0) that facilitates the degradation of ILVs and their cargo by activating lysosomal hydrolases [81,82]. These enzymes degrade the cargo within ILVs into their basic components, such as fatty acids, amino acids, and nucleotides. The degradation products are recycled for metabolism or excreted, maintaining cellular homeostasis by preventing waste accumulation [83]. The final escape opportunity involves lysosomal membrane permeabilization, a process that compromises the lysosomal membrane integrity and releases mRNA into the cytoplasm [84]. However, lysosomal membrane permeabilization risks cellular homeostasis, as the leakage of lysosomal hydrolases and protons into the cytosol damages cellular components and triggers cell death or inflammation [85]. Therefore, inducers that can cause the permeability of the lysosomal membrane require cautious use to avoid cytotoxicity. Fortunately, cells employ natural repair mechanisms for lysosomal damage. The repair process involves the recruitment of the ESCRT machinery to constrict and invaginate damaged sites, the supply of new lipids through phosphoinositide signaling and lipid transfer, and the annexin-mediated membrane resealing [86].

### 2.4. Potential Strategies for Enhancing mRNA Escape from Endo-Lysosome System

The potential strategies for enhancing mRNA escape from the endo-lysosomal system are embedded within the intracellular journey of mRNA-loaded LNPs, which is summarized in Table 1. Upon cellular uptake, the initial route of mRNA loss occurs during the early endosomal stage, where it is recycled to the extracellular environment via the apparatus of the recycling endosome. Manipulating this process can enhance mRNA intracellular delivery, as disrupting endosomal recycling reduces mRNA expulsion. For example, Xu, J. et al. reported that reducing recycling endosomes can be effectively achieved by targeting SNX16, a crucial sorting nexin. They found that silencing SNX16 disrupts normal recycling trafficking, leading to a decrease in E-cadherin recycling to the cell surface [87]. Similarly, depleting the deubiquitylating enzyme USP32 inhibits late endosome cargo recycling, causing accumulation and swelling of late compartments, which may be a potential way to reduce recycling function [88]. According to the mechanisms of endocytic recycling, enhancing retromer function, utilizing SNX27, leveraging retriever complexes, and promoting WASH complex-driven recycling are effective strategies to minimize cargo expulsion. Forming tubular carriers with BAR domain proteins and facilitating recycling via EHD1 and ACAP1 also play crucial roles. However, impairing the recycling endosome pathway risks cargo accumulation, reduced nutrient uptake, and disrupted receptor signaling, therefore compromising cellular homeostasis [72].

The early endosomal stage is also the primary site for natural endosomal escape. Nucleic acids are theorized to occur via LNP–endosomal membrane fusion [89]. Promoting this fusion is the most important strategy for enhancing mRNA escape. Ionizable lipids within the LNP formulation drive the fusion events. As endosome pH drops below the apparent pKa, protonation induces a positive charge. This facilitates electrostatic interactions with anionic endosomal lipids, triggering a bilayer to hexagonal phase transition that enables membrane fusion and cytosolic cargo release. Experimental evidence supports the critical role of ionizable lipids in this process [90,91]. For instance, ionizable lipids with pKa in the range of 6.2–6.5 effectively facilitate endosomal escape and the subsequent cytosolic delivery of nucleic acids [92]. Studies utilizing LNPs containing lipids such as DLin-MC3-DMA or SM-102 demonstrate high gene expression via protonation-driven fusion [80,93]. Furthermore, an endosomal membrane mimic-dependent study demonstrated the pH-dependent fusion peaks at 6.6 to 6.0. Notably, the fusion efficiency is influenced by the LNP formulation, with higher DSPC concentrations correlating with lower fusion efficiency. These findings underscore the LNP formulation optimizing for endosomal escape enhancement [94].

Lysosomal membrane permeabilization represents the final escape opportunity before degradation. There are several molecules have been investigated to mediate the escape of cargo by lysosomal membrane permeabilization [95]. Photo-induced escape by photosensitizer is the main way to achieve this goal. The near-infrared light irradiation of porphyrin-cooperated LNPs generates reactive oxygen species (ROS), disrupting lysosomal membranes and doubling RNA release [96]. Based on this theory, PCI Biotech developed a new technology that exhibits notable efficacy in head and neck squamous cell carcinoma. A photothermal reaction was also employed to mediate the lysosome permeabilization. Upon NIR irradiation, the photothermal sensitizer generated heat, therefore disrupting endosomal membranes and facilitating RNA delivery into the cytoplasm [97]. Additionally, the proton sponge effect, which involves the buffering capacity of polycations like polyethyleneimine (PEI), can also promote endosomal escape. Recent studies showed that PEI could buffer the acidic environment of endosomes, potentially leading to osmotic swelling and rupture, although this effect may not be the sole mechanism for endosomal escape [98,99]. Recently, a study found that the addition of metal ions like manganese in LNPs enhances the lysosomal escape of mRNA by improving the pH buffering capacity of the nanoparticles [100]. In fact, any agents that could induce lysosomal membrane permeabilization can facilitate mRNA escape.

## 3. Progress of LNPs for mRNA Endo/Lysosomal Escape

The endosomal/lysosomal escape performance of LNPs is rooted in the properties of their fundamental components or unique physical modifications, while LNPs typically comprise four core components: cationic/ionizable lipids, phospholipids, cholesterol, and polyethylene glycol-ylated lipids (PEG-lipids). Cationic/ionizable lipids are required to form electrostatic interactions with negatively charged mRNA, facilitating its encapsulation. Phospholipids and cholesterol contribute to the formation and stabilization of LNPs; they help to maintain the structural integrity and facilitate membrane fusion processes. PEGylated lipids form a hydrophilic stealth coating that prolongs circulation half-life and enhances cellular uptake [101]. Overall, each of these four components confers distinct yet synergistic roles in LNP formation, stability, and endosomal escape functionality. Extensive literature demonstrates that each segment of the LNP component can be strategically optimized for specific biological applications, where minor molecular alterations yield substantial improvements in endo/lysosomal escape performance. In this section, we systematically review these advancements with particular emphasis on component-level innovations that promote endo/lysosomal escape.

### 3.1. Cationic/Ionizable Lipid Molecular Engineering

As the most scrutinized components, the structural optimization of cationic lipids has undergone progressive refinement. Wei, Y. and his colleagues engineered a series of amphiphilic cationic lipids by systematically altering the lengths (C = 8–18), quantities (*n* = 2, 4), and degrees of unsaturation (Ω = 0, 1) of their hydrophobic tails. Notably, their newly synthesized lipid–mRNA formulations achieved efficient nucleic acid compaction, protection, and release, indicating that the length of the hydrophobic tails is a key factor in determining the formation and stability of the lipid assemblies. At a specific length, the presence of unsaturated tails enhances the membrane fusion and fluidity of the assemblies, thereby significantly influencing transgene expression. While the number of hydrophobic tails also plays a role to a lesser extent [102]. A fluorescence resonance energy transfer (FRET)-based assay further demonstrated that the unsaturated citronellol tail, 4A3-Cit, exhibits superior lipid fusion capabilities due to its disruptive unsaturated structure. This promotes mRNA release into the cytoplasm, boosting delivery efficacy [103]. However, the permanent positive charge led to significant toxicity and limited their clinical applications [104,105].

To address this, the backbone structure of DLin-MC3-DMA was modified by incorporating alkyne and ester groups into the lipid tails. The performance of these modified lipids was assessed when co-formulated with other amine-containing lipid-like materials. Notably, the introduction of alkyne lipids significantly enhanced membrane fusion, thereby enhancing mRNA release and resulting in a synergistic improvement in mRNA delivery efficiency [106]. This discovery was further extended by a newly identified ionizable lipid, U19, with significant endo/lysosomal escape and prolonged mRNA expression duration compared to ALC-0315, synthesized from a series of ionizable lipids with two, three, and four tails and characterized, featuring an imidazole group as the head [107]. Mrksich, K. et al. have well reviewed the role of the structure of cationic and ionizable lipids in LNPs escaping the endosome, emphasizing the impact of lipid tails, linkers, and cores on endosomal escape mechanisms. Future works should refine these structures for effective LNP-mediated nucleic acid delivery [90].

Accompanying the optimization of the structure, stimuli-responsive bivalent lipids have emerged. For example, the pH-dependent behavior of ionizable cationic lipids like DODMA is vital in endosomal escape. At low pH, DODMA interacts weakly with RNA, anchoring it near the lipid bilayer surface. As pH rises, this interaction becomes repulsive, causing RNA to redistribute from lipid headgroup regions to the hydrophilic slabs of the multilayers within the lipoplex. DODMA also increased leaflet flip at high pH to facilitate fusion with the endosomal membrane. These changes improve mRNA delivery and therapeutic efficacy [108]. In a recent study, a library of stimuli-responsive bivalent ionizable lipids was synthesized to enhance mRNA vaccine delivery and immune response. These lipids, featuring a degradable scaffold, two amine heads, and a hydrophobic lipid tail, were designed to degrade rapidly in response to intracellular cues such as esterase, H_2_O_2_, cytochrome P450, alkaline phosphatase, nitroreductase, or glutathione. Specifically, the esterase-responsive lipid (eBiv iLP) rapidly degrades in antigen-presenting cells to drive robust antigen-specific CD8^+^ T cells to infiltrate tumors and orchestrate innate and adaptive immunity to control tumor growth [109]. Collectively, these advances underscore the critical role of cationic/ionizable lipids in mediating the LNP endosomal escape.

### 3.2. Helper Lipid Innovations

**Figure 3:**
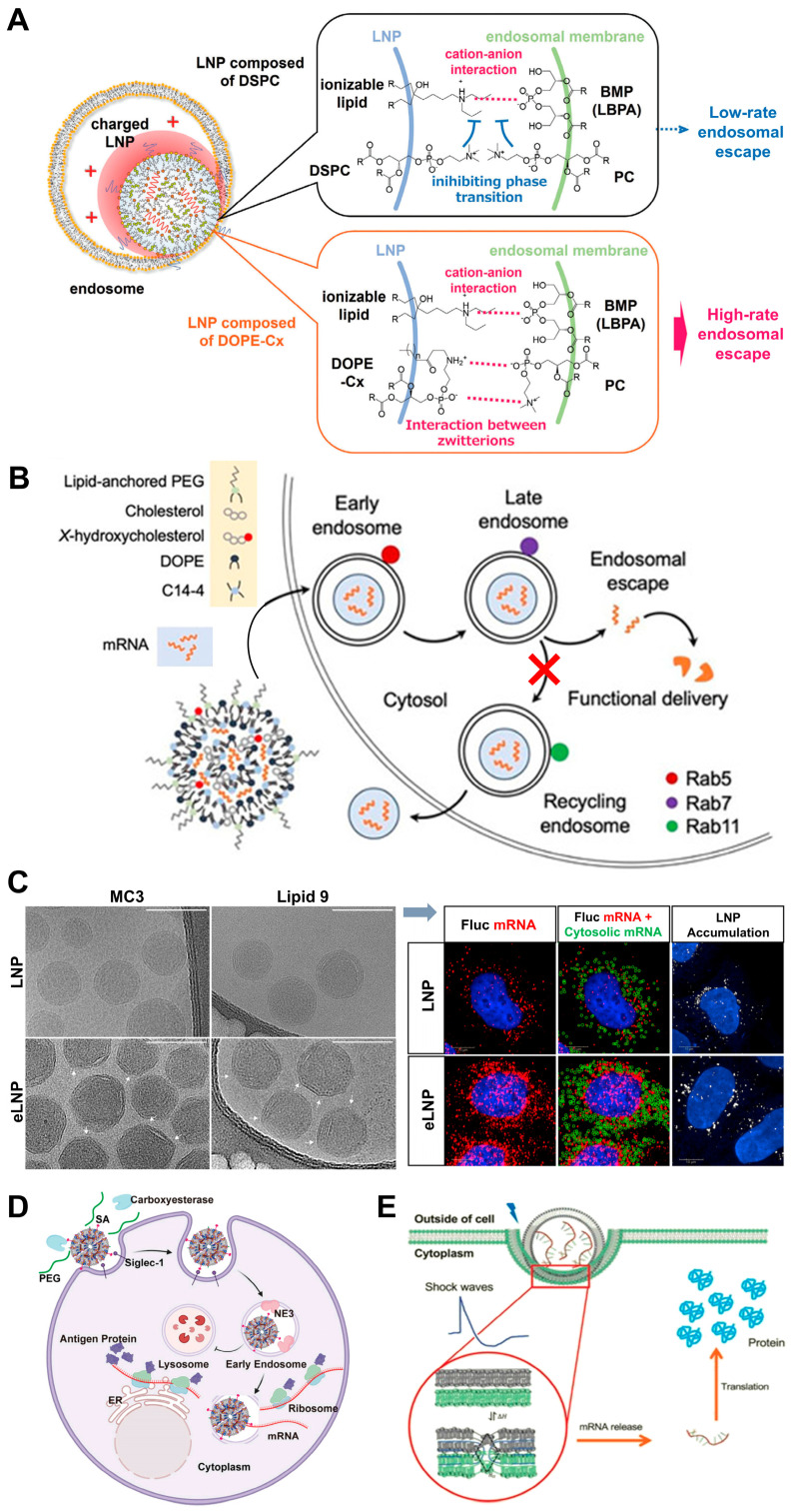
Representative research of the endo/lysosomal-escapable LNPs for mRNA delivery. (**A**) For LNPs composed of DOPE-Cx, the interaction between PC in the endosomal membrane and DOPE-Cxs forms ionic pairs, which disrupts the stability of the lamellar phase of DSPC and facilitates a phase transition. Ultimately, this phase transition promotes the endosomal escape of the encapsulated mRNA. Copyright 2025 by Wiley. (**B**) Replacing 25% and 50% of cholesterol with 7α-hydroxycholesterol in LNPs results in a diminished number of recycling endosomes and an increased production of late endosomes. This compositional adjustment enhances the delivery of mRNA to primary human T cells in ex vivo experiments. Copyright 2022 by Elsevier. (**C**) Cryo-TEM observed distinct structural variations between normal LNPs and the incorporation of β-sitosterol into LNPs (eLNP), Scale = 100 nm. While monitoring the endosomal escape by smFISH, the authors obtained representative fluorescent images showing mRNA and LNPs and conducted image analysis after delivery with LNPs or eLNPs in HeLa cells, Scale = 10 μm. For higher clarity, please refer to the original publication. Copyright 2020 by Nature Publishing Group. (**D**) The schematic representation of the transfection mechanism by SA-modified LNPs indicates that the encapsulated mRNA is released into the cytoplasm upon its exit from the early endosomes, circumventing lysosomal degradation. Furthermore, mRNA within the early endosome may be diverted toward the Golgi apparatus and endoplasmic reticulum pathways. Copyright 2023 by Elsevier. (**E**) The acoustically responsive fusogenic LNP demonstrated enhanced delivery when the transfection occurred in the presence of acoustic shock waves. For higher clarity, please refer to the original publication. Copyright 2019 by ACS Publications.

Among the four key components of LNPs, phospholipids have been relatively overlooked in both fundamental and translational research. To explore the role of phospholipids, their potential was investigated by systematically modifying the phospholipid component within LNPs and identifying delivery efficiency. The results demonstrated that the chemical nature of phospholipids can significantly influence mRNA delivery by promoting membrane fusion and facilitating endosomal escape. Notably, phospholipids with phosphoethanolamine (PE) head groups appear particularly effective in enhancing endosomal escape, likely due to their inherent fusogenic characteristics [110]. Expanding this concept, Sagi, A. et al. also studied the impact of integrating bis (monoacylglycerol) phosphate (BMP) lipid into LNPs and assessed the in vivo functional transport of mRNA by these formulated nanoparticles. Their findings revealed that LNPs incorporating BMP enhanced endosomal membrane fusion and more efficient delivery of mRNA to the cytosol [111]. A novel zwitterionic phospholipid (DOPE-Cx), by attaching hydrophobic moieties to the zwitterionic head group of DOPE, was found to alter its orientation and interact more effectively with phosphatidyl choline (PC). PC is a major component of cellular membranes, and this modification overcomes PC’s inhibitory effect on lipid-phase transitions. Specifically, DOPE-Cx can form ion pairs with PC in the endosomal membrane to facilitate a phase transition from a lamellar phase to a non-lamellar cubic phase. This transition promotes membrane fusion, allowing mRNA to be released from endosomes into the cytosol more efficiently. The study demonstrates that DOPE-Cx, particularly those with shorter hydrophobic chains (such as DOPE-C8), readily induce the formation of cubic phases when mixed with PC. This property enhances endosomal escape efficiency and improves the functional delivery of mRNA (Figure 3A) [112]. With the advances of photo-responsive systems, Li, B. et al. synthesized Cy-lipid via nucleophilic substitution between a thiol lipid and NIR-II dye. Cy-lipid was also incorporated into LNPs with SM-102, cholesterol, and DMG-PEG2000 to encapsulate mRNA. In acidic endosomes, protonated Cy-lipid activates NIR-II absorption, converting 1064 nm laser energy into heat. This heat induces morphological changes in NIR-II LNPs, disrupting the endosomal membrane and enabling mRNA to escape into the cytosol. Cellular experiments showed a 3-fold increase in eGFP mRNA translation upon laser irradiation. In vivo studies in mice demonstrated a 4.5-fold boost in liver luciferase mRNA translation efficiency. This strategy effectively enhances mRNA delivery and protein production by leveraging Cy-lipid’s photothermal effects to trigger endosomal escape [113].

### 3.3. Cholesterol Optimization Strategies

Cholesterol plays a pivotal role in modulating the physicochemical properties and biological functions of LNPs, especially in providing structural integrity and influencing membrane fluidity [114]. Therefore, cholesterol and its analogs have also become a target for improving the efficiency of endosomal escape. Recently, Patel, S.K. et al. reported that LNPs containing 25% and 50% substitutions of 7α-hydroxycholesterol show increased co-localization with acidic organelles, indicating enhanced endosomal accumulation. However, these modified LNPs also reduce the expression of Rab11, the marker for recycling endosomes, thereby decreasing endosomal recycling and increasing the likelihood of endosomal escape. Consequently, the hydroxycholesterol substitution in ionizable LNPs significantly enhances mRNA delivery to T cells by altering endosomal trafficking mechanisms (Figure 3B) [115]. In contrast, the C-24 substitution of cholesterol with naturally occurring analogs can significantly promote the endosome membrane fusion process. Through a series of experiments, Siddharth Patel et al. discovered that those with C-24 alkyl substitutions, such as β-sitosterol, significantly outperformed traditional cholesterol-based LNPs in mRNA transfection, with up to 211-fold improvement in transfection efficiency (Figure 3C) [77]. The cryo-transmission electron microscopy, small-angle X-ray scattering, and 3D single-particle tracking showed that the β-sitosterol-containing eLNPs with polyhedral, faceted shapes have a more regular lamellar phase with tighter lipid, mRNA packing and improved intracellular delivery. The underlying principle was attributed to the C-24 alkyl group in β-sitosterol introducing minor defects in the lipid bilayer organization of the LNPs. These defects might facilitate membrane destabilization, promote fusion with endosomal membranes, and enable the efficient release of mRNA into the cytosol [77]. Experimental evidence showed that LNP-Sito showed a 10-fold increase in detectable late endosomal perturbation events when compared to the standard cholesterol LNPs, suggesting the superior capability of LNP-Sito to escape from endosomal entrapment [116]. In addition to their unique morphology, Sito-LNPs demonstrate a high degree of multi-lamellarity, with approximately 45% of the particles forming multiple lipid bilayers. This multilamellar structure is believed to enhance gene delivery by allowing multiple fusion events with the endosomal membrane, thereby increasing the likelihood of successful mRNA release into the cytosol. The presence of these multilamellar structures also suggests a higher degree of lipid packing and rigidity, which might be an advantage to maintaining structural integrity during cellular uptake and transit through the endosomal pathway [117]. Based on these findings, researchers have explored the use of corosolic acid derivatives to replace cholesterol in LNPs. These cholesterol-free and corosolic acid-containing LNPs (CAxLNPs) demonstrated significantly improved cellular uptake and endosomal escape. Fluorescence resonance energy transfer assays and hemolysis experiments revealed that CAxLNPs had a great capacity for membrane fusion compared to traditional cholesterol LNPs. The enhanced fusion ability allows for more efficient release of mRNA from endosomes into the cytoplasm, thereby improving transfection efficiency [118]. On the contrary, the absence of cholesterol in the sodium alginate-coated LNP formulation allows for easier fusion of DOTAP molecules with the lysosomal membrane, further enhancing escape efficiency [119].

### 3.4. PEG Lipid Engineering

The type and content of PEGylated lipids significantly influence mRNA-LNP endosomal escape. In vitro studies revealed that LNPs containing DMG-PEG2k with a shorter acyl chain exhibited superior endosomal escape compared to those with longer acyl chains like DSG-PEG2k and DSPE-PEG2k. This enhancement stems from the faster shedding rate of DMG-PEG2k from the nanoparticle structure, facilitating quicker mRNA release and translation. Similarly, in vivo experiments confirmed that LNPs with DMG-PEG2k showed higher transfection efficiency, attributable to their enhanced endosomal escape capability. In addition, the content of PEGylated lipids is equally crucial. LNPs with 1.5 mol% PEGylated lipids exhibited optimal transfection efficiency in both in vitro and in vivo settings. Lower contents compromised the stability of the LNPs, while higher contents hindered mRNA release, thus reducing endosomal escape efficiency. These findings highlight the importance of selecting PEGylated lipids with appropriate acyl chain lengths and optimizing their concentration to maximize endosomal escape and overall transfection efficiency [120]. Besides the type and content optimization, structure modification further enhances LNPs’ performance. Zhang, H. et al. developed a fluorinated modification of PEG-DSPE (termed FPD). In their study, the FPD, accounting for only 1.5% of the lipids in LNPs, could induce a substantial improvement in mRNA expression efficiency in both tumor cells and primary dendritic cells (DCs). Mechanistic studies revealed that FPD-LNPs were able to escape from lysosomes and uniformly distribute in the cytoplasm within 4 h of treatment, whereas non-fluorinated LNPs remained largely colocalized with lysosomes [121]. Acid-responsive groups like the azido-acetal linker were also used to synthesize acid-degradable PEG lipids, which were then incorporated into LNPs to create ADP-LNPs. The ADP-LNPs maintained high levels of PEGylation extracellularly, enhancing their circulation time and diffusion through tissues. Upon endocytosis, the PEG chains were rapidly hydrolyzed in the endosomes, promoting fusion with the endosomal membrane and facilitating endosomal escape. In vitro experiments showed that ADP-LNPs with high PEG content (up to 40 mol%) efficiently transfected cells, whereas conventional LNPs with non-degradable PEG at similar levels were ineffective. The enhanced endosomal escape was confirmed through fluorescent microscopy using Galectin-8 reporter cells [79].

### 3.5. Surface Coating and Shape Management

Beyond component-level structures, surface engineering methods demonstrate synergistic effects. Pardaxin has been reported to mediate the enhanced transfection efficiency by facilitating the non-lysosomal delivery route, therefore reducing the lysosome degradation of cargo. Yu Liu et al. developed a pardaxin-modified liposome, namely P-Lipo, and found that P-Lipo could reduce the degradation of mRNA by lysosomes through a non-lysosomal intracellular pathway, significantly improving the transfection efficiency of mRNA in DCs compared with unmodified liposomes [122]. They further modified LNPs using coiled-coil lipopeptides and found the formation of coiled-coil structures between these lipopeptides not only accelerated nucleic acid uptake but also significantly enhanced mRNA expression. The cellular uptake of these coiled-coil-modified LNPs was predominantly driven by membrane fusion, therefore bypassing conventional endocytic pathways. This cytosolic delivery route eliminates endosomal entrapment limitations [123].

For escape, a study developed SA-modified mRNA-loaded LNPs that simultaneously achieved DC targeting and efficient endosomal escape. The SA modification promoted the rapid uptake of LNPs by DCs and enabled over 80% of early endosomes to escape within 2 h, avoiding entry into lysosomes. Specifically, the co-localization of SA-modified LNPs with lysosomes was less than 10%, while other LNPs showed co-localization rates of 40% to 60% at 120 min. This efficient endosomal escape significantly enhanced the expression of target proteins in DCs. In tumor-bearing mouse models, SA-modified LNPs demonstrated superior antitumor efficacy compared to commercially formulated mRNA vaccines (Figure 3D) [124]. Another study explored the use of sodium alginate-coated cationic mRNA LNPs (SA@DOTAP-mRNA) to enhance endosomal escape and improve tumor immunotherapy. Specifically, SA@DOTAP-mRNA nanoparticles exhibit a low co-localization rate with endo/lysosomes, indicating their efficient escape from the endosomal pathway. This is mainly attributed to the neutralization of H^+^ ions by SA side chains, which allows the uncharged hydrophobic side chains to insert into the hydrophobic part of the endosomal membrane, reducing endosomal stability and facilitating escape. In terms of tumor treatment, SA@DOTAP-mRNA demonstrates superior antitumor efficacy compared to conventional cationic liposome/mRNA complexes, which can be reflected by the tumor growth inhibition and the established tumor regression [119].

The shape of LNPs also significantly regulates endosomal escape. LNPs can form various shapes such as lamellar, inverse hexagonal, and bicontinuous cubic phases. Among them, bicontinuous cubic and inverse hexagonal structured LNPs, like cuboplexes, show enhanced fusogenicity with endosomal membranes. Results demonstrate that these non-lamellar LNPs can more readily fuse with endosomes. This leads to a higher extent of endosomal escape, as shown by more efficient RNA delivery and less endosomal entrapment in cells compared to lamellar-structured LNPs [125]. Accordingly, the internal structure and surface properties of LNPs are also influencing the efficiency of endosomal escape. Research has demonstrated that the internal structure of mRNA-loaded LNPs is characterized by a disordered inverse hexagonal phase, which is independent of LNP size but is more pronounced in the presence of mRNA. This structure facilitates the release of mRNA from the LNP core, allowing it to diffuse out upon fusion with the endosomal membrane. The findings suggest that optimizing the size and surface characteristics of LNPs can significantly enhance intracellular protein production, as larger LNPs with a specific surface composition showed higher transfection efficacy [126].

### 3.6. Ancillary Enhancement Strategies

The combination use of function-specific drugs can precisely help the mRNA-loaded LNPs. To address the challenge of mRNA efficacy erosion in the endosome–lysosome pathway, two potent small molecules, NAV2729 (NAV) and endosidin 5 (ES5), were identified to markedly augment the delivery efficiency of mRNA by LNPs. Mechanism exploration revealed that NAV inhibits ARF6-dependent endocytic recycling, while ES5 could disrupt the interaction of Annexin A6 (ANXA6) with lipids, thereby promoting mRNA release. NAV and ES5 do not accelerate the uptake of mRNA-loaded LNPs but rather facilitate the release of mRNA into the cytoplasm by targeting recycling endosomes. These findings underscore the potential of targeting endosomal recycling as a strategy to potentiate mRNA-loaded LNP delivery, highlighting the distinct mechanisms involved in mRNA release compared to siRNA delivery [127]. Similarly, Patel, S. et al. discovered that the mTOR signaling pathway, localized on lysosomes, plays a key role in regulating mRNA translation. By screening bioactive lipid-like molecules, they found that leukotriene antagonists, such as MK-571, significantly boosted intracellular mRNA delivery both in vitro and in vivo. Their work indicates the potential of using the drug-based modulation of cellular signaling pathways to improve LNP-mediated mRNA delivery for therapeutic applications [128].

Novel physico-chemical synergies are expanding conventional LNP design paradigms. More recently, the thermodynamic state of LNPs has been highlighted in enhancing mRNA delivery. The optimized LNP through acoustically responsive design features two phase transitions near physiological conditions. These transitions are key to overcoming the energy barrier for fusion, enabling efficient mRNA endosomal escape. Shock waves can enhance endosomal escape by promoting phase transitions and fusion, significantly increasing mRNA expression (Figure 3E) [129]. Chen, J. et al. demonstrated that mechanical oscillation at a frequency of 65 Hz could significantly enhance the endosomal escape of mRNA encapsulated in LNPs by promoting the fusion of oppositely charged LNPs. The enhanced fusion facilitates the release of mRNA from endosomes into the cytoplasm, thereby improving transfection efficiency. Importantly, the mechanical oscillation does not compromise cell viability or cause significant damage to cellular structures, such as the mitochondrial membrane potential and Golgi apparatus integrity [130].

## 4. Summary and Outlook

The employment of mRNA in cancer therapeutics has been extraordinary in recent years, establishing a transformative technological foundation for precision oncology. LNPs have played an indispensable role in advancing mRNA delivery to target cells, yet the bottleneck of internalized mRNA cargo being entrapped within endo/lysosomal vesicles, ultimately undergoing enzymatic degradation, persists. Given that cytoplasmic mRNA liberation is a prerequisite for ribosomal translation, enhancing intracellular delivery efficiency has emerged as the central challenge in therapeutic mRNA applications. These challenges are driving intensive research on engineering LNPs with endo/lysosomal escape capabilities. Recent innovations in lipid component optimization, nano-structural control, and stimulating drug combinations are redefining the boundaries of mRNA bioavailability. The relative advancements represent not merely a technical refinement, but a pivotal strategy to unlock the full clinical potential of mRNA cancer therapies.

The intracellular pathways through which mRNA-loaded LNPs traverse, including phagocytosis, macropinocytosis, CME, and CVME, significantly determine not only the initial uptake efficiency but also the subsequent trafficking dynamics and opportunities for endosomal escape. Given that most of those pathways are involved in the endosomal–lysosomal transport system, CVME offers a potential pathway to bypass lysosomal degradation. CVME can internalize nucleic acids and transport them to the Golgi apparatus and endoplasmic reticulum, thus avoiding the acidic and degradative environment of lysosomes. This pathway leverages the natural trafficking pathways of caveolae, enhancing the bioavailability of nucleic acids and reducing their degradation [131].

Following endocytosis, enhancing the probability of mRNA to escape endo/lysosomal compartments can be achieved in three processes: reducing recycling endosome-mediated efflux, promoting natural endosomal escape, and inducing lysosomal membrane permeabilization. Each process can leverage specific lipid components within the LNP formulation to optimize mRNA delivery. Membrane fusion-mediated natural endosomal escape is currently the predominant mechanism, with the majority of studies supporting that structural and compositional adjustments of the LNP components, including cationic/ionizable lipids, phospholipids, cholesterol, and PEG-lipids promote the fusion of LNPs with endosomal membranes, thereby mediating mRNA escape. These investigations have identified some commonalities for better membrane fusion properties in lipid molecular structures. It should be emphasized that beyond the basic components of LNPs, the spatial arrangement affected by lipids and RNA interactions significantly influences the efficiency of escape in the membrane fusion process [132]. In addition, while the method for inducing lysosomal membrane permeabilization has been extensively studied, most of them are not fundamental components of LNPs [133,134]. For example, recent studies have reported that the manganese addition could promote mRNA escape in lysosomes [135]. However, since these substances are not integral parts of LNPs, they are not included in this review. It is worth noting that these established strategies for modifying carriers and the active molecules capable of inducing lysosomal membrane permeabilization hold promise for application in mRNA-loaded LNP systems. However, permeabilizing the lysosomal membrane may not be an ideal approach for mRNA escape, as it has the likelihood to undermine cell viability and impede subsequent translation processes [136]. Importantly, the direct inhibition of recycling endosomes via lipid molecules is not currently feasible, but the combination of mRNA-loaded LNPs with drugs that inhibit recycling endosomes holds great promise for enhancing mRNA escape.

Numerous studies have successfully designed LNP carriers to facilitate mRNA endo/lysosomal escape over the past decade. The advantages of these carriers with endosomal/lysosomal escape capabilities are clearly compared in Table 2. However, our review of the pathways of mRNA-loaded LNP internalization and the processes of mRNA escape from the endosome–lysosome system reveals limitations in the current understanding. A major issue is that many studies have not made a clear distinction between endosomes and lysosomes, leading to ambiguity in the precise procedure of mRNA escape. This lack of differentiation hinders the clarity of escape mechanisms and underscores the need for more precise delineation of the endo/lysosomal system in future studies. Such differentiation would aid in guiding further optimization of LNPs. Additionally, while various experimental methods have been employed to observe the escape events, there is a notable absence of a universally accepted quantitative standard for measuring escape efficiency. This standard is crucial for evaluating final efficacy, as recent research has demonstrated that mRNA escape is not always directly proportional to protein expression [137]. Therefore, establishing a robust quantitative metric for escape could provide more accurate insights into the therapeutic potential of mRNA-loaded LNPs.

Following the escape, there are still unknown factors regarding the translation efficacy issue, which significantly impact the therapeutic effect of mRNA after its escape. Studies have shown that the expression levels of mRNA delivered by LNPs are significantly influenced by cellular oxygen levels, with hypoxic conditions leading to reduced protein expression after mRNA escape from endosomes. This limitation is closely associated with decreased intracellular ATP concentrations under hypoxia. Supplementing ATP within the LNP formulations has been shown to enhance mRNA expression levels by a 79-fold increase in vitro and a 24-fold increase in vivo, demonstrating that ATP is a key factor in modulating the efficacy of mRNA delivery [138,139]. Research in this area will be a strong supplementary support for endo/lysosomal-escapable LNP platforms in mRNA delivery.

LNPs with endo/lysosomal escape capabilities in cancer therapy hold immense promise. By engineering LNPs to effectively liberate mRNA from endosomal and lysosomal compartments, we can expect more robust and sustained protein expression, which is crucial for applications such as cancer immunotherapy, tumor suppressor restoration, and precise genetic modifications within cancer cells. Additionally, exploring the combination of endosomal escape-enhancing LNPs with other therapeutic modalities, such as immunomodulatory agents or chemotherapeutics, will open new frontiers in multimodal cancer treatment strategies.

## Figures and Tables

**Table 1:** Potential strategies for enhancing mRNA escape from the endo-lysosomal system.

Key Steps	Challenges	Strategies	References
Sorting of LNPs/mRNA within early endosomes.	A significant portion of internalized LNP/mRNA (~70% for siRNA) can be recycled back out of the cell via recycling endosomes, reducing the intracellular dose.	Inhibit or modulate endocytic recycling pathways: Block key proteins Target recycling machinery components Modulate retromer, SNX27, retriever, and WASH complexes.	[72,75,77,87,88]
	Impairment of recycling pathways can disrupt cellular homeostasis.	Use interventions with caution, considering potential side effects on normal cellular functions.	[72]
Release of mRNA from endosomes (early or Rab11-positive recycling endosomes) into the cytoplasm.	Low natural efficiency of endosomal escape for conventional LNPs. Most mRNA remains trapped and gets degraded.	Enhance LNP-endosomal membrane fusion: Utilize pH-responsive ionizable lipids (pKa ~6.2–6.5) that become cationic in acidic endosomes. Optimize LNP formulation (lipid composition, DSPC concentration).	[76,78,79,80,89,90,91,92,93,94]
	Escape is influenced by endosomal properties (acidification, size, type).	Target escapes from favorable endosomal compartments Ensure endosomal acidification for ionizable lipid function.	[78]
Potential release of mRNA from lysosomes into the cytoplasm.	Lysosomes exhibit substantial degradative functions. The induction of lysosomal membrane permeabilization (LMP) presents a potential risk of cytotoxicity and subsequent cell death.	Induce controlled LMP through photo-induced methods, photothermal techniques, and the proton sponge effect, such as those involving PEI and manganese.	[84,85,86,95,96,97,98,99,100]
	Cellular mechanisms exist to repair lysosomal damage.	Formulate strategies that either minimally activate apoptotic pathways or utilize intrinsic cellular repair mechanisms.	[85,86]

**Table 2:** Advancements of endo/lysosomal-escapable LNP platforms for enhancing mRNA delivery.

Innovations	Mechanisms	Efficacy	References
Cationic amphiphilic lipids with systematically varied hydrophobic tail lengths, quantities, and degrees of unsaturation	Length of hydrophobic tails determines formation and stability; unsaturated hydrophobic tails enhance membrane fusion and fluidity.	Achieved efficient nucleic acid compaction, protection, and release; significantly influenced transgene expression.	[102]
Cationic lipid with an unsaturated citronellol tail (4A3-Cit)	Unique unsaturated structure promotes better disruption and fusion with endosomal membranes.	Demonstrated superior lipid fusion capabilities; enhanced fusion ability facilitates mRNA release from endosomes into the cytoplasm, improving overall efficacy.	[103]
Lipids with alkyne and ester groups incorporated into the DLin-MC3-DMAbackbone structure	Introduction of alkyne lipids significantly enhanced membrane fusion.	The release of mRNA is enhanced, leading to a synergistic improvement in the efficiency of mRNA delivery.	[106]
New identified ionizable lipid, U19 (from a series with two, three, and four tails featuring an imidazole head group)	Significant endo/lysosomal escape.	Significant endo/lysosomal escape, prolonged mRNA expression duration compared to ALC-0315.	[107]
Ionizable cationic lipids like DODMA	At low pH, DODMA interacts weakly with RNA, anchoring it near the lipid bilayer surface. As the pH increases, this interaction becomes repulsive, leading to the redistribution of RNA within the lipoplex. Additionally, DODMA enhances leaflet flipping at high pH to facilitate fusion with the endosomal membrane.	Alterations in pH facilitate endosomal escape, thereby enhancing mRNA delivery and therapeutic efficacy.	[108]
Esterase-responsive bivalent ionizable lipid LNPs	Designed to degrade rapidly in response to intracellular cues; rapid degradation of LNP in antigen-presenting cells, leading to efficient mRNA release.	Efficient mRNA release, robust antigen presentation; induced high magnitude of antigen-specific CD8+ T cells to infiltrate tumors and orchestrate innate and adaptive immunity to control tumor growth.	[109]
LNPs with systematically modified phospholipid component	Chemical nature of phospholipids can significantly influence mRNA delivery by promoting membrane fusion and facilitating endosomal escape; PE head groups are effective due to inherent fusogenic characteristics.	Significantly influence mRNA delivery, enhance endosomal escape.	[110]
LNPs incorporating bis (monoacylglycerol) phosphate (BMP) lipid	Enhanced endosomal membrane fusion.	More efficient delivery of mRNA to the cytosol.	[111]
Novel zwitterionic phospholipid (DOPE-Cx, e.g., DOPE-C8) by attaching hydrophobic moieties to the zwitterionic head group of DOPE	Alters orientation and interacts more effectively with phosphatidyl choline (PC); forms ion pairs with PC in the endosomal membrane to facilitate a phase transition from a lamellar phase to a non-lamellar cubic phase, promoting membrane fusion.	Enhances the efficiency of endosomal escape and improves the functional delivery of mRNA; DOPE-C8 readily induces cubic phases when mixed with PC.	[112]
NIR-II LNPs (incorporating Cy-lipid synthesized via nucleophilic substitution between a thiol lipid and NIR-II dye)	In acidic endosomes, protonated Cy-lipid activates NIR-II absorption, converting 1064 nm laser energy into heat, inducing morphological changes, disrupting the endosomal membrane, and enabling mRNA escape.	3-fold increase in eGFP mRNA translation upon laser irradiation (cellular); 4.5-fold boost in liver luciferase mRNA translation efficiency (in-vivo); effectively enhances mRNA delivery and protein production.	[113]
LNPs containing 25% and 50% substitutions of 7α-hydroxycholesterol	Increased co-localization with acidic organelles (enhanced endosomal accumulation); reduces expression of Rab11, decreasing endosomal recycling and increasing the likelihood of endosomal escape by altering endosomal trafficking mechanisms.	Significantly enhances mRNA delivery to T cells.	[115]
LNPs with C-24 alkyl substitutions of cholesterol (e.g., β-sitosterol, LNP-Sito)	C-24 alkyl group introduces minor defects in the lipid bilayer organization, facilitating membrane destabilization and promoting fusion with endosomal membranes. LNP-Sito has polyhedral, faceted shapes, a more regular lamellar phase with tighter lipid/mRNA packing, and a high degree of multi-lamellarity (45% particles forming multiple lipid bilayers), allowing multiple fusion events.	Significantly outperformed traditional cholesterol-based LNPs in mRNA transfection, with up to 211-fold improvement in transfection efficiency; 10-fold increase in detectable late endosomal perturbation events; superior capability to escape from endosomal entrapment; enhanced gene delivery.	[77,116,117]
Cholesterol-free and corosolic acid-containing LNPs (CAxLNPs)	Great capacity for membrane fusion.	Significantly improved cellular uptake and endosomal escape; improved transfection efficiency.	[118]
SA@DOTAP-mRNA formulation (absence of cholesterol)	Absence of cholesterol allows for easier fusion of DOTAP molecules with the lysosomal membrane.	Enhanced escape efficiency.	[119]
LNPs containing DMG-PEG2k (with shorter acyl chain)	Faster shedding rate of DMG-PEG2k from the nanoparticle structure, facilitating quicker mRNA release and translation.	Superior endosomal escape compared to longer acyl chains (DSG-PEG2k and DSPE-PEG2k); higher transfection efficiency (in vivo); optimal transfection efficiency at 1.5 mol% PEGylated lipids.	[120]
Fluorinated modification of PEG-DSPE (FPD) in LNPs	FPD-LNPs escape from lysosomes and uniformly distribute in the cytoplasm within 4 h; accelerates the release of mRNA from endosomes.	Substantial improvement in mRNA expression efficiency in both tumor cells and primary DCs; increased efficiency of mRNA transfection and expression.	[121]
Acid-degradable PEG lipids (ADP-LNPs) synthesized with azido-acetal linker	Maintained high levels of PEGylation extracellularly (enhancing circulation/diffusion); rapid hydrolysis of PEG chains in endosomes upon endocytosis, promoting fusion with the endosomal membrane and facilitating endosomal escape.	Efficiently transfected cells with high PEG content (up to 40 mol%) where conventional LNPs were ineffective; enhanced endosomal escape confirmed.	[79]
Pardaxin-modified liposomes (P-Lipo)	Facilitates non-lysosomal delivery route, reducing lysosome degradation of cargos.	Reduced degradation of mRNA by lysosomes; significantly improved the transfection efficiency of mRNA in DCs compared with unmodified liposomes.	[122]
LNPs modified using coiled-coil lipopeptides	Formation of coiled-coil structures accelerates nucleic acid uptake; cellular uptake predominantly driven by membrane fusion, bypassing conventional endocytic pathways, leading to direct delivery into the cytosol.	Significantly enhanced mRNA expression; effectively circumvented the common issue of limited endosomal escape.	[123]
Sialic acid (SA)-modified mRNA loading LNPs	Promoted rapid uptake by DCs; enabled over 80% of the LNPs to escape from early endosomes within 2 h, avoiding entry into lysosomes (co-localization with lysosomes less than 10% vs. 40–60% for others).	Efficient endosomal escape crucial for successful mRNA translation, significantly enhancing target protein expression in DCs; superior antitumor efficacy compared to commercially formulated mRNA vaccines in tumor-bearing mouse models.	[124]
Sodium alginate (SA)-coated cationic mRNA LNPs (SA@DOTAP-mRNA)	Low co-localization rate with endo/lysosomes; neutralization of H^+^ ions by SA side chains allows uncharged hydrophobic side chains to insert into the hydrophobic part of the endosomal membrane, reducing endosomal stability and facilitating escape.	Efficient escape from the endosomal pathway; superior antitumor efficacy compared to conventional cationic liposome/mRNA complexes (tumor growth inhibition, established tumors regression).	[119]
Non-lamellar LNPs (e.g., bicontinuous cubic and inverse hexagonal structured LNPs, cuboplexes)	Enhanced fusogenicity with endosomal membranes; more readily fuse with endosomes.	Higher extent of endosomal escape, more efficient RNA delivery, and less endosomal entrapment compared to lamellar structured LNPs.	[125]
mRNA-loaded LNPs with disordered inverse hexagonal phase internal structure	Structure facilitates the release of mRNA from the LNP core, allowing it to diffuse out upon fusion with the endosomal membrane.	Optimizing size and surface characteristics can significantly enhance intracellular protein production; larger LNPs with a specific surface composition showed higher transfection efficacy.	[126]
LNP-mRNA (optimized by molar ratio of ionizable lipids to mRNA nucleotides)	A 1:1 molar ratio of ionizable lipids to mRNA nucleotides is optimal; at this ratio, the mRNA is neutrally charged by the ionizable lipids, facilitating its translocation across the negatively charged endosomal membrane.	Optimal for enabling mRNA to escape from the endosomal membrane into the cytoplasm.	[75]
mRNA-loaded LNPs combined with small molecules NAV2729 (NAV) and endosidin 5 (ES5)	NAV inhibits ARF6-dependent endocytic recycling; ES5 disrupts the interaction of Annexin A6 (ANXA6) with lipids; both facilitate mRNA release by targeting recycling endosomes without accelerating LNP uptake.	Markedly augment the delivery efficiency of mRNA by LNPs; potentiate mRNA-loaded LNPs delivery.	[127]
LNP-mediated mRNA delivery combined with leukotriene antagonists (e.g., MK-571)	Leukotriene antagonists modulate the mTOR signaling pathway.	Significantly boosted intracellular mRNA delivery both in vitro and in vivo.	[128]
Acoustically responsive fusogenic LNP	Features two phase transitions near physiological conditions to overcome the energy barrier for fusion; shock wave promotes phase transitions and fusion.	Enables efficient mRNA endosomal escape and protein expression; significantly increasing mRNA expression; enhanced delivery when transfection occurred in the presence of acoustic shock waves.	[129]
LNP-mediated mRNA delivery enhanced by mechanical oscillation	Mechanical oscillation at a frequency of 65 Hz significantly enhances the endosomal escape by promoting the fusion of oppositely charged LNPs; enhanced fusion facilitates the release of mRNA from endosomes into the cytoplasm.	Significantly enhanced endosomal escape; improved transfection efficiency; does not compromise cell viability or cause significant damage to cellular structures.	[130]

## Data Availability

The dataset can be downloaded from the Web of Science core collection according to the retrieval criteria mentioned in the manuscript.
